# Deep Unsupervised Learning on a Desktop PC: A Primer for Cognitive Scientists

**DOI:** 10.3389/fpsyg.2013.00251

**Published:** 2013-05-06

**Authors:** Alberto Testolin, Ivilin Stoianov, Michele De Filippo De Grazia, Marco Zorzi

**Affiliations:** ^1^Computational Cognitive Neuroscience Lab, Department of General Psychology, University of PadovaPadova, Italy; ^2^IRCCS San Camillo Neurorehabilitation HospitalVenice Lido, Italy

**Keywords:** deep neural networks, unsupervised learning, hierarchical generative models, cognitive modeling, parallel-computing architectures, GPUs, MPI, computer cluster

## Abstract

Deep belief networks hold great promise for the simulation of human cognition because they show how structured and abstract representations may emerge from probabilistic unsupervised learning. These networks build a hierarchy of progressively more complex distributed representations of the sensory data by fitting a hierarchical generative model. However, learning in deep networks typically requires big datasets and it can involve millions of connection weights, which implies that simulations on standard computers are unfeasible. Developing realistic, medium-to-large-scale learning models of cognition would therefore seem to require expertise in programing parallel-computing hardware, and this might explain why the use of this promising approach is still largely confined to the machine learning community. Here we show how simulations of deep unsupervised learning can be easily performed on a desktop PC by exploiting the processors of low cost graphic cards (graphic processor units) without any specific programing effort, thanks to the use of high-level programming routines (available in MATLAB or Python). We also show that even an entry-level graphic card can outperform a small high-performance computing cluster in terms of learning time and with no loss of learning quality. We therefore conclude that graphic card implementations pave the way for a widespread use of deep learning among cognitive scientists for modeling cognition and behavior.

## Introduction

Unsupervised learning in neural network models has provided important insights into how sensory information can be efficiently encoded in neural systems in a way that strikingly mirrors single-cell recoding data (Olshausen and Field, 1996; Rao and Ballard, 1999). Much recent work has tackled the issue of how sensitivity to increasingly complex features might emerge from unsupervised learning in a hierarchical architecture, under the assumption that hierarchical processing is a fundamental characteristic of cortical computation (Hinton, 2007; Clark, 2012). This approach, known as “deep learning” (Bengio, 2009), is based on multilayer neural networks that learn a generative model of the sensory data without supervision and it is attractive both from a machine learning (Hinton and Salakhutdinov, 2006; Bengio and Lamblin, 2007; Larochelle et al., 2009; Lee et al., 2009; Mohamed et al., 2012; Yu et al., 2012) and from a cognitive (neuro)science perspective (Hinton, 2007; Honglak et al., 2008; Stoianov and Zorzi, 2012). Probabilistic generative networks learn an internal model of the world from sensory signals and actively use this knowledge to infer causes and make predictions about relevant events (Hinton and Ghahramani, 1997; Hinton, 2010b; Huang and Rao, 2011; Clark, 2012). Within this framework, perception is formulated as probabilistic inference on the input data, given a set of hidden causes learned from statistical regularities inherent in the observed world.

Deep unsupervised learning has several advantages over traditional neural network learning schemes. Hierarchical, deep networks consist of a composition of non-linear processing stages that transforms the incoming information into higher-level representations at each step and allows the system to capture higher-order structure that might be invisible at the lower levels (Hinton and Ghahramani, 1997). Learning in a deep belief network can be seen as fitting a hierarchical generative model to the sensory data, where learning aims at reconstructing the input data from the internal representations and can be performed locally at each level in an unsupervised fashion. This represents a novelty in training multilayer neural networks, because it demonstrates how probabilistic learning can be performed using a mechanism that is both efficient (Hinton and Osindero, 2006) and neurally plausible (O’Reilly, 1998). Deep unsupervised learning can capture most of the statistical structure in the input data and it represents an efficient coding strategy. For these reasons, it holds the promise of improving our understanding of the complexity of information representation in neural systems as well as of capturing key aspects of human behavior within an emergentist framework (Stoianov and Zorzi, 2012). Moreover, deep unsupervised learning can properly exploit the huge set of unlabeled information that is available to the learner in the environment, thereby dispensing with the psychologically implausible assumption that learning must be driven by an external teacher [note that this assumption is ill-founded even for complex learning tasks such as reading aloud; for discussion see (Share, 1995; Zorzi, 2010)]. Importantly, building higher-level abstractions can reveal causal features that are only implicit in the sensory data [for example, the numerosity of visual sets in the model of Stoianov and Zorzi (2012)] and which may allow subsequent task learning (e.g., identification, categorization, or other types of judgments) through simple linear mappings. Finally, unsupervised pre-training of deep belief networks is also a promising strategy to improve supervised (Hinton and Salakhutdinov, 2006) and transfer learning (Pan and Yang, 2010; Bengio, 2011).

Training large deep networks is computationally intensive, because it requires huge datasets and the optimization of millions of model parameters (i.e., synaptic weights) (Hinton and Salakhutdinov, 2006). Moreover, though very simple (“toy”) models can provide important insights into the neural bases of cognition, large-scale simulations allow researchers to investigate the emergence of more complex phenomena (Le et al., 2012; Stoianov and Zorzi, 2012). Modern computer architectures provide the computational resources required for scaling up the neural network modeling of perception and cognition. For example, high-performance parallel computing (HPC) in combination with efficient learning algorithms has been recently exploited for object classification using a multilayer neural network with a billion connections using a dataset of 10 million images (Le et al., 2012). Even at a somewhat smaller scale, the use of HPC may be necessary for developing large-scale simulations in cognitive psychology and cognitive neuroscience. Indeed, one approach to efficiently simulate neural network models includes the use of multi-core clusters (Plesser et al., 2007), usually exploiting Message Passing Interface (MPI) communication (Margaris et al., 2007; De Filippo De Grazia et al., 2012b). Support for HPC is also available in some neural network simulators (e.g., Aisa et al., 2008; Mutch et al., 2010).

Since the introduction of CUDA, which is a massive parallel-computing framework for common graphic processor units (GPUs) presented by NVIDIA, many computational tasks can be efficiently carried out on graphic cards (Nickolls et al., 2008). Deep learning algorithms largely involve simple matrix manipulations and are therefore well suited to be implemented on graphic processors. Machine learning researchers are already adopting this emerging technology for training deep networks because it yields an impressive speed-up of training time, thereby allowing scaling up the size of both network and training database (e.g., Raina et al., 2009; Ciresan et al., 2010). In particular, GPU computing has been recently used to train very large feed-forward neural networks for image processing using the classic backpropagation algorithm (Ciresan et al., 2010), also with convolutional layers (Krizhevsky et al., 2012). Deep unsupervised learning was instead implemented by Raina et al. (2009) on GPUs using fine-grained CUDA programming to train one of the largest deep belief networks to date. Nevertheless, the fact that the use of deep neural networks and deep unsupervised learning is still largely confined to the machine learning community suggests that cognitive scientists might have been discouraged by the lack of a user-friendly implementation as well as the high computational demands of the simulations.

In the present work, we present a straightforward GPU implementation of deep belief networks trained with contrastive divergence learning (Hinton and Salakhutdinov, 2006) that is based on high-level programming routines and can run on a common desktop computer, provided that it has a recent NVIDIA graphic card. We also performed an empirical comparison between our GPU implementations and a multi-core CPU implementation (De Filippo De Grazia et al., 2012b) running on a small HPC cluster with up to 60 cores. Our open-source codes for all parallel implementations (based on MATLAB/Octave or Python) are publicly available for download (see Appendix for details). We found that even an entry-level GPU significantly outperforms the cluster with respect to the computational time required for deep unsupervised learning, with no cost on the quality of learning. We believe that this ease of use, combined with the low cost of powerful graphic cards, will allow non-expert users to explore deep generative networks and boost research on this promising approach to modeling cognition and behavior.

## Unsupervised Learning with Deep Belief Networks

**Figure 1:**
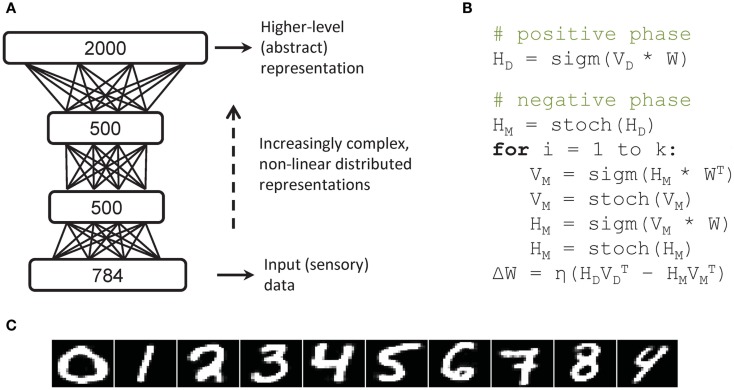
**Deep network used as a test bed problem for comparison of parallel implementations**. **(A)** Structure of the deep network composed of a stack of three Restricted Boltzmann Machines (RBM), whereby input consists of vectorized images and is provided to the lowest layer. **(B)** Pseudocode of the learning algorithm of one RBM layer, which computes contrastive divergence with *k* iterations. **(C)** Sample digit-image reconstructions.

In this work we considered the same network architecture used by Hinton and Salakhutdinov (2006), depicted in Figure 1A. The model is composed by a stack of three Restricted Boltzmann Machines (RBMs) (Smolensky, 1986; Hinton, 2002), which are stochastic neural networks that consist of one layer of visible units (input data) and one layer of hidden units (latent causes of the data) connected by symmetric links. Boltzmann Machines are associative neural networks in which an “energy” function defines the probability of all configurations of visible and hidden units (Ackley et al., 1985). RBMs can be trained to learn a generative model of the sensory data: learning is unsupervised because the objective function is to accurately reconstruct the input patterns. One efficient learning algorithm for RBMs is contrastive divergence (Hinton, 2002), which is a form of contrastive optimization that approximates the gradient of the log-likelihood of the learning data. Essentially, it works by clamping the visible units on a given training instance to compute hidden units activation (*positive phase*) and then running a Markov Chain Monte Carlo algorithm in order to obtain the model’s reconstruction of that instance (*negative phase*). Because RBMs have no intra-layer connections (unlike full Boltzmann Machines), the sampling process can be speeded-up by performing block Gibbs sampling over visible and hidden units (i.e., all units in a layer can be sampled in a single step). However, running a Markov chain until convergence may still require an exponential time, hence contrastive divergence learning approximates the gradient by performing only a fixed number *k* of iterations. After computing the model’s reconstruction, weights are updated by contrasting input-output correlations computed on the data vector with input-output correlations computed on the model’s reconstruction,

$$\Delta W=\eta \left(v^{+}h^{+}-v^{-}h^{-}\right)$$

where η is the learning rate, *v*^+^ indicates visible activations clamped on a training instance, *h*^+^ are hidden activations computed from *v*^+^, while *h*^−^ and *v*^−^ are visible and hidden activations sampled from the model’s distribution.

A crucial feature of this learning algorithm is that it only requires very simple matrix operations, as outlined in the pseudocode reported in Figure 1B, where *H*_D_ and *V*_D_ represent, respectively, hidden and visible units when the network is clamped on input data, *H*_M_ and *V*_M_ represent the model’s reconstructions and *W* is the matrix of connection’s weights. The *stoch* operator performs a stochastic binarization of a vector, while *sigm* indicates the logistic function,

$$sigm\left(z\right)=\frac{1}{1+e^{-z}}$$

The pseudocode in Figure 1B highlights that contrastive divergence learning requires a heavy, iterative use (depending on the parameter *k*) of basic mathematical operators that can be applied in an element-wise fashion. This is exactly what is needed in order to obtain maximum performances on graphic processors.

The recommended way to train RBMs using the contrastive divergence algorithm is to split the entire dataset into smaller, non-overlapping subsets, called *mini-batches* (Hinton, 2010a). Instead of iteratively updating the network weights with the gradient computed on each training pattern (“on-line learning”) or rather updating with the average gradient computed across the entire dataset (“off-line learning”), the gradient in mini-batch learning is averaged over the patterns of the mini-batch. This improves convergence and learning speed by both varying and smoothing the learning gradient (Wilson and Martinez, 2003).

The Appendix provides download links and instructions for using our open-source codes implementing unsupervised training of deep belief networks on different parallel-computing architectures. We offer a MATLAB and a Python solution for GPUs, as well as an Octave/MPI cluster solution. The codes are fairly general and can be easily used on any learning problem. We also provide instructions for testing the algorithm on two different problems: handwritten digit recognition [MNIST dataset; (LeCun and Bottou, 1998)], a classic benchmark used in machine learning, and visual numerosity perception (Stoianov and Zorzi, 2012) as a sample cognitive modeling problem.

## Simulations

As a benchmark for our parallel implementations of deep belief networks we used a classic vision problem, handwritten digit recognition. The hierarchical processing in biological vision systems suggests that successful solutions would greatly benefit from the extraction of invariances at multiple levels, departing from the sensory input (Hinton, 2007; Stoianov and Zorzi, 2012). Indeed, deep neural networks can discover such processing hierarchies. Following Hinton and Salakhutdinov (2006), we trained a deep network to accurately generate the images of handwritten digits presented during learning. In the original study, deep unsupervised learning on the unlabeled images was followed by a supervised learning phase. That is, the image labels (10 classes, one for each digit) were used to fine-tune the network weights for the digit recognition task using error backpropagation. The supervised phase can be useful for better discriminative learning and it might be necessary for achieving state-of-the-art performance in comparison to other machine learning algorithms. However, from a cognitive modeling perspective, fine-tuning with error backpropagation is not necessary (Stoianov and Zorzi, 2012) and it might be unwarranted due to the well-known implausibility of the algorithm (for discussion, see O’Reilly, 1998). Accordingly, we measured and compared processing times during the unsupervised deep learning phase only. The fine-tuning procedure was still carried out in order to assess the quality of the learned model, measured in terms of classification accuracy. We also directly tested the quality of the internal representations that emerged from deep unsupervised learning to exclude any confound introduced by the supervised fine-tuning phase. To this aim, we trained a linear classifier to recognize the digit class using the top-level hidden layer representation as input (see De Filippo De Grazia et al., 2012a; Stoianov and Zorzi, 2012;, for applications of this method to cognitive neuroscience modeling). Learning was performed by computing a direct solution using the pseudo-inverse method (Hertz et al., 1991). Classification errors on a separate test set were collected and averaged over 10 different networks for each type of parallel implementation.

### Network architecture and training dataset

As in the original study (Hinton and Salakhutdinov, 2006), we used a network with three hidden layers (500–500–2000 units, respectively, for a total of about 1.6 million connections) trained using one-step contrastive divergence (CD-1). As training data we used the popular MNIST dataset (LeCun and Bottou, 1998) that contains handwritten digits encoded as 28 × 28-pixel gray-level images, size-normalized, mass-centered, and manually classified. The dataset contains 60,000 training images and 10,000 test images. Hinton and Salakhutdinov (2006) showed that deep learning on these images produces rich internal representations of the digits that can readily be exploited for recognition, yielding state-of-the art performance in terms of classification error. As in the original study, the models we obtained were able to accurately reconstruct input images (see samples in Figure 1C).

### Parallelization

A parallelization at the *data-level* implies distributed and parallel processing of multiple training patterns across different computing units. A prerequisite for this type of parallelization is that learning is not fully iterative, but the weight updates are calculated across multiple patterns as in off-line or mini-batch learning. In a cluster implementation, the patterns of the training set (or the subset of patterns that form a mini-batch) are distributed across the available CPU cores. Weight updates are computed at each core with respect to the assigned data-packet and subsequently averaged to obtain the final weight update for the network. This strategy requires relatively little communication between nodes, which is a critical constraint on cluster architectures. In contrast, the implementation on GPUs can also take advantage of a *layer-level* parallelization, which implies parallel processing for multiple neurons. That is, the activation of each neuron can be carried out by a separate graphic core. Thus, on GPUs we can combine layer-level and data-level parallelization by manipulating 2-D matrices that contain multiple patterns. In other words, we can perform element-wise matrix operations in parallel, with almost no communication delay if all data can be loaded into the GPU’s memory. The enormous computing power of GPUs derives from their internal architecture that exploits a great number of simple cores that operate in parallel. This hardware design is well suited to efficiently process graphic information (e.g., real-time rendering of visual scenes by mapping textures and applying lightening to geometric shapes), which is usually represented using matrices of items that can be manipulated element-wise. The basic idea that allowed to exploit GPUs for scientific computing was that here too we often have to apply simple functions to a large number of elements at the same time (Owens and Houston, 2008).

#### Graphic processors

The implementation on graphic processors is straightforward. All we need to do is to load the training dataset into the GPU’s memory and to adapt the source code to specify which operations have to be performed on the graphic card. One possibility is to directly use CUDA routines, which allow a fine-grained control of the parallelization by specifying data distribution and threads synchronization, as well as an optimized allocation of the GPU’s memory hierarchy (e.g., Raina et al., 2009; Ciresan et al., 2010). We instead exploited high-level wrappers of CUDA available in MATLAB via the Parallel-Computing Toolbox (version 2011 or higher) (Sharma and Martin, 2008) and in Python via the Gnumpy module (Tieleman, 2010). This choice was motivated by the fact that the flow of sensory processing is almost linear and inherently parallel. The use of such high-level wrapper functions greatly simplified the parallelization, which only required the use of *gpu array* data types instead of standard arrays and feeding the graphic processors with mini-batches. Differently from a cluster implementation (see below), here we did not have to explicitly control the distribution of each mini-batch onto the graphic cores, because the parallelization is made transparent to the user by the high-level libraries. Since the original source code (including the fine-tuning phase) and all the routines used for the analyses were written in MATLAB, we only present results for that GPU implementation. Nevertheless, we verified that the Python code for deep unsupervised learning had similar performance^1^.

One bottleneck in GPU computing is the relatively slow transfer of data between central memory and the GPU. We optimized the algorithm by using single- instead of double-precision data types, which allowed us to load the whole training dataset (200 MB) and network into the GPU, and perform the entire learning there with minimal CPU-GPU communication^2^.

#### Multi-core cluster

We used the most common parallel programing paradigm for computer clusters, which is message passing. The messages transport data and synchronize the independent calculations. In particular, we used MPI, which is a language-independent communication protocol, extending the original code (Hinton and Salakhutdinov, 2006) by adding collective MPI routines.

In our cluster implementation, a master-node distributes the processing of each mini-batch by splitting it between the available cores (slave-nodes). The master node also transmits to each core the current state of the network weights. The slave-nodes independently compute the vectors of weight updates according to their specific data-packet. The updates proposed by each core are then collected by the master-node and averaged in order to calculate the new weights of the network, which are then distributed back to all nodes for the processing of the following mini-batch (De Filippo De Grazia et al., 2012b). In the original study of Hinton and Salakhutdinov (2006), the size of the mini-batch was fixed to 125 patterns. We chose this value as size of the data-packet processed by a single core. As more cores are available, weight updates are computed over many such data-packets in parallel and then averaged. This corresponds to using mini-batches of size S = *k* × 125, where *k* is the number of cores. That is, on 2, 4, 8, 16, 40, and 60 cores this leads to mini-batches of 250, 500, 1000, 2000, 5000, and 7500 patterns.

### Hardware details

The GPU implementations were tested on two different graphic cards. One was an NVIDIA GeForce GTX 460 (Fermi architecture) equipped with 336 CUDA cores (1.35 GHs) and 1 GB of DDR5 dedicated memory. The other was an NVIDIA GeForce GTX 690 (Kepler architecture), for a total number of 1536 CUDA cores (1.41 MHz) and 2 GB of DDR5 memory^3^.

The cluster implementation was executed on a HP distributed computing cluster composed of seven nodes, each equipped with quad-core or hexa-core processors (2.27 GHz) and 32 GB of RAM. Overall, there were 60 cores. The nodes were interconnected with Infiniband technology network^4^. The cluster was controlled by Linux, Octave 3.0.5, and the Open-MPI library^5^. Open-MPI routines were executed from Octave through the MPITB toolbox^6^. This toolbox implements all necessary point-to-point and collective MPI communication routines needed, and it has been shown to outperform other MPI toolboxes for Octave/MATLAB (Fernández et al., 2006).

As a baseline, we also collected running times on a PC workstation equipped with an Intel Q6600 quad-core CPU (2.40 GHz), controlled by Linux and Octave 3.2.

## Results

### Computational time for deep unsupervised learning

**Figure 2:**
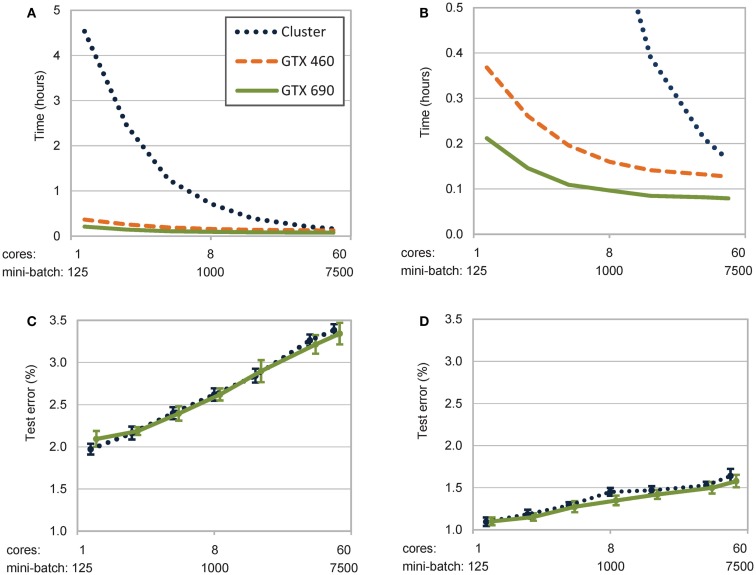
**Trade-off between learning times and learning quality as a function of mini-batch size (abscise) on a log scale**. **(A)** Unsupervised leaning time on all parallel architectures decreases with mini-batch size. The greater the number of patterns simultaneously processed, the more the computational resources involved (e.g., processing cores). **(B)** Zoom-in of learning times highlighting the additional speed-up of the GTX 690 card. **(C)** Quality of learning on the cluster and on the GTX 690 implementations, measured as misclassification of a linear classifier trained on the top-layer internal representations. **(D)** Quality of learning after fine-tuning of the entire deep model (see text for details).

A comparison between learning times for the different implementations is shown in Figure 2A (means and standard deviations are reported in Table 1). The most impressive result is the substantially lower computational time required by GPUs. On low and medium mini-batch sizes (e.g., 125, 250, 500, and 1000, which on the cluster correspond to using 1, 2, 4, and 8 cores), even the entry-level GTX 460 outperformed the cluster implementation by one order of magnitude. The high-performance GTX 690 card further improved the result (as highlighted in Figure 2B), requiring a learning time that was half that of the cluster also on the largest mini-batch size (i.e., 7500, which corresponds to using 60 cores). Notably, learning times on GPUs were between 11 and 45 times faster than on the quad-core PC.

**Table 1:** **Unsupervised learning times for various mini-batch sizes on a PC workstation, GPUs (MATLAB implementation), and a computer cluster**.

	Learning time (s) ± SD			
Mini-batch size	Quad-core	GTX 690	GTX 460	Cluster (overhead)
125	15191 ± 3	764 ± 7	1337 ± 24	16330 ± 430 (05%)
250	14184 ± 7	526 ± 4	895 ± 14	8828 ± 173 (11%)
500	13679 ± 5	393 ± 9	665 ± 27	4493 ± 485 (17%)
1000	13043 ± 3	348 ± 16	528 ± 5	2590 ± 83 (27%)
2000	12746 ± 12	306 ± 15	450 ± 6	1411 ± 52 (28%)
5000	12832 ± 18	294 ± 5	429 ± 13	757 ± 7 (33%)
7500	12934 ± 21	285 ± 7	417 ± 11	590 ± 3 (44%)

*The rightmost column reports the communication overhead (in percentage) on the computer cluster*.

The significant difference between the cluster and GPU implementations reflects the advantage of exploiting both data- and layer-level parallelization on GPUs, which can optimally use the available computational resources even when only few data patterns are to be processed at once. It should be pointed out that these improvements were obtained using *k* = 1 for *CD-k* learning, and it is not to be excluded that using higher values of *k* would further improve the GPU’s speed-up.

Notably, all simulations exhibited exponential speed-up with the increase of mini-batch size, as shown in Figure 2A and Figure 2B. A power fit analysis revealed that the cluster implementation speeded-up fastest with the increase of mini-batch size (power coefficient *k* = −0.82, *r*^2^ = 0.99), while the GPU implementations showed less-pronounced improvements (GTX460: *k* = −0.25, *r*^2^ = 0.90; GTX690: *k* = −0.22, *r*^2^ = 0.86). In the cluster implementation, the asymptotic behavior for large mini-batch sizes (and hence, more computing cores) is due to increasing overhead caused by data transport and synchronization. In order to estimate the impact of this overhead, we profiled the code by calculating the ratio of the time spent to collect and send data relative to the overall running time. The results reported in the rightmost column of Table 1 show that the percentage of time spent for data transfer increased along with mini-batch size.

The results demonstrate that the parallelization on the computer cluster is faster for large mini-batches, that is when more cores are independently processing distinct subsets of training patterns. Indeed, on very large mini-batches, the cluster performance approaches that of GPUs. Unfortunately, this also causes a decrease of learning quality, which is reflected by the lower classification accuracy reached by models trained using large mini-batch sizes, as we discuss below.

### Classification error after deep unsupervised learning

We first evaluated the quality of the internal representations obtained after unsupervised learning only, by training a linear classifier using the top-level (2000 units) hidden layer representations of the input data. As hinted in Section “Unsupervised Learning with Deep Belief Networks,” the more iterative is the learning, the less prone it is to fall into local minima despite that it is a coarser approximation of the true gradient of the error function (Wilson and Martinez, 2003). We therefore expected that by increasing mini-batch size, classification accuracy would get worse. This was confirmed by analysis of classification error.

Results are shown in Figure 2C. Analysis of variance on the accuracy data (within-subject factor: mini-batch size, *n* = 7; between-subject factor: parallel implementation type, *n* = 2) revealed a significant effect of mini-batch size [*F*(6,108) = 598.5, *p* < 0.001] but no effect of implementation type [*F*(1,18) = 1.3] or their interaction [*F*(6,108) = 2.0]. Linear regression analysis with mini-batch size as a predictor (on a log scale) confirmed the prediction above that the classification error increases along with mini-batch size (slope = 0.77, *r*^2^ = 0.99). These results also demonstrate that using a single-precision floating-point format on GPUs did not affect the quality of the learning process.

### Classification error after supervised fine-tuning

To provide a link with the machine learning literature on this benchmark visual recognition task, we also report the classification performance after an additional fine-tuning of the entire network with backpropagation. To avoid potential confounds, the fine-tuning phase was performed on a single core.

As shown in Figure 2D, both implementations obtained comparable classification performance after the fine-tuning phase, although the same analysis of variance performed in Section “Classification Error After Deep Unsupervised Learning” revealed some advantage for the GPU implementation [*F*(1,18) = 39.0, *p* < 0.001]. The effect of mini-batch size was also significant [*F*(6,108) = 177.5, *p* < 0.001]. The two-way interaction was not significant [*F*(6,108) = 1.5]. Also here a linear regression analysis demonstrated that the classification error after fine-tuning increases with mini-batch size (slope = 0.28, *r*^2^ = 0.98), though in this case the effect of mini-batch size was less pronounced (as shown by the smaller slope of the regression function).

## Discussion

Deep belief networks (Hinton and Salakhutdinov, 2006) efficiently extract high-level internal representations of input data by learning hierarchical generative models in an unsupervised fashion. Their layered structure is inspired by the hierarchical organization of cortical networks and their functioning has a sound probabilistic formulation (Hinton, 2007). For these reasons they are particularly appealing for developing neural models of cognition, as shown in a recent work on modeling numerosity perception (Stoianov and Zorzi, 2012). However, deep unsupervised learning is also computationally very demanding, especially if we want to scale-up to more realistic simulations. Modern parallel-computing architectures have been proven to be effective for training large-scale deep networks, reaching impressive performance over various machine learning benchmarks like visual object recognition and speech recognition (Ciresan et al., 2010; Krizhevsky et al., 2012; Le et al., 2012; Mohamed et al., 2012). Unfortunately, expertise in parallelization techniques and expensive hardware may not be available to many cognitive scientists and this might have hindered the diffusion of deep belief networks as a tool for connectionist modeling of cognition and behavior.

The purpose of this paper was twofold. We first presented a ready-to-use parallel implementation of a deep belief network, which exploits the power of modern graphic cards (GPUs) and can therefore run on standard desktop PCs. Parallelization on GPUs was obtained using high-level software libraries available for two of the most popular programing languages used in scientific computing, namely MATLAB and Python. We then compared the GPU implementation with another based on a multi-core HPC cluster. We used as a benchmark for our simulations a deep network with 1.6 million connections and a popular learning task (LeCun and Bottou, 1998) that involves the extraction of statistical regularities from a large dataset of 60,000 handwritten digit images.

Both implementations exhibited an exponential reduction of learning times when using mini-batches of increasing size. However, learning large mini-batches comes at the cost of a lower classification accuracy, which indexes a lower quality of the learned models. These results highlight a trade-off between learning times and learning quality and, accordingly, a clear advantage in using GPUs. Indeed, on small and medium mini-batch sizes, which yield higher learning quality, the GPUs reached a speed-up of more than an order of magnitude with respect to the computer cluster. Moreover, with respect to a stand-alone quad-core PC, the speed-up reached a 45-fold increase. This impressive performance confirms the value of graphic cards for deep unsupervised learning (Raina et al., 2009), because the underlying code involves an intensive use of basic matrix operations and functions (Owens and Houston, 2008). Neural network learning can be parallelized at different levels, the most intuitive of which distributes the processing of different neurons among different nodes (layer-level parallelization). However, layer-level parallelization involves intensive communication between nodes, which is very time-consuming on a cluster implementation. Data-level parallelization, which consists in distributing all training patterns or subsets of them (mini-batches) among the available cores, is instead effective because between-core communication is minimal (De Filippo De Grazia et al., 2012b). The appeal of a GPU implementation is that both levels of parallelization can be effortlessly combined by adopting a high-level programing platform and a parallel-computing toolbox, which optimally distributes the calculations among the available graphic resources.

It is well-known that iterative learning (i.e., “on-line learning”) approximates the true gradient of the error function, but it is less prone to fall into local optima than optimizing the error function on the entire dataset (Wilson and Martinez, 2003). However, iterative learning requires more computations (weight updates). Iterative learning of mini-batches is a compromise between those two. Although there are some empirical suggestions about how to choose mini-batch sizes for training deep belief networks (Hinton, 2010a), we are unaware of a systematic investigation of the effect of this parameter on the quality of the learned model. We thus exploited a wide range of mini-batch sizes, expecting that their increase would be associated with increase of classification error, that is, worsening the obtained statistical model of the sensory data. However, small mini-batches need more frequent updates of model parameters that must be synchronized across the processors. In the cluster implementation, the relatively slow between-core communication would therefore cause a strong trade-off between learning speed-up and accuracy of classification. The results confirmed the predictions. Smaller mini-batches systematically led to better models but required longer learning times. These trends were found on all parallel implementations. Cluster times were heavily penalized for small batches, which suggests using medium-sized mini-batches (e.g., 1000 patterns) on such implementation. In contrast, the substantially lower learning times for all mini-batch sizes obtained by the GPU implementation permits using small mini-batches (i.e., 125 elements), thus yielding the best-quality models. In other words, the GPU implementation allows one to target the highest levels of accuracy with only a moderate loss in learning speed. Finally, it is worth noting that the models obtained with different parallel implementations reached almost the same classification performance when tested either right after the unsupervised learning or after an additional supervised fine-tuning phase (as in the original work of Hinton and Salakhutdinov, 2006) with a slight advantage of the GPU parallelization. Overall, the results suggest a net advantage of the GPU implementation.

Although the benefit of using GPUs for computational purposes has been shown in several domains, it should be stressed that their successful application heavily depends on the nature of the computations involved in the problem of interest (Lee et al., 2010). The possibility to load the entire dataset on the GPU memory is important to obtain high performance. In some situations this might not be possible, with the risk of introducing significant delays when frequently transferring data between the central memory (RAM) and the GPU. Single GPUs, due to their somewhat limited memory, are thus not suitable for scaling up to very large models trained on extensive datasets, and other parallelization approaches based on large HPC clusters or supercomputers might be necessary (Dean et al., 2012; Le et al., 2012). A promising approach would be to combine the power of multiple GPUs in a very large-scale distributed architecture.

It is worth noting that the aim of this study was neither to obtain the fastest training on GPUs nor to claim that cluster implementations are not suited to compete with GPUs. Instead, our goal was to emphasize that deep belief networks, which represent the state-of-the-art in neurocognitive modeling (Stoianov and Zorzi, 2012) and engineering applications (Mohamed et al., 2012) can obtain excellent performance on common graphic hardware with little programing efforts. Adapting the learning algorithm to run on a graphic card was straightforward and it did not involve specific issues concerning the engineering of the parallelization. In contrast, the implementation on a computer cluster posed constraints on the level of parallelization, which was feasible only at the data-level (De Filippo De Grazia et al., 2012b) and, as we demonstrated above, was associated with an important time-quality trade-off. Note that our results should generalize also to more recent deep generative network architectures (e.g., Deep Boltzmann Machines, Salakhutdinov and Hinton, 2012). The source codes for deep unsupervised learning provided in Appendix are not tied to the specific benchmark task and they can be easily adapted to other learning problems. We provide a variety of solutions (MATLAB/Octave on a desktop PC; MATLAB and Python on a desktop with graphic card; Octave/MPI on a multi-core cluster) and we leave it to individual researchers to choose the one that best fits their needs. Interested users can try these methods on the MNIST database that we used here as benchmark problem as well as on the dataset of images used by Stoianov and Zorzi (2012) for deep learning of numerosity perception (see Appendix for instructions).

A final aspect that deserves attention regards the economic cost of the hardware required by our parallel implementations. The price of an entry-level graphic card is about 100€, while the cost of a 60-nodes computer cluster exceeds 30,000€. Moreover, the Python solution provides a freeware implementation of deep unsupervised learning on graphic cards. In conclusion, parallelization of deep belief networks on GPUs using high-level languages can bring medium-scale simulations on a desktop computer at a very affordable cost and with no time investment on acquiring parallel programing skills.

## Conflict of Interest Statement

The authors declare that the research was conducted in the absence of any commercial or financial relationships that could be construed as a potential conflict of interest.

## Footnotes

^1^The Python implementation actually turned out to be faster than the MATLAB implementation. Note that all components of the Python implementation are freeware.

^2^Since the cluster implementation used the standard double-precision data types, to which the CPU is optimized, we ensured that running times on the cluster were not affected by the choice of double vs. single-precision values.

^3^The GTX 690 is a dual-graphic card with two distinct sets of graphic processors (i.e., 1536 cores and 2 GB memory for each set). We used only one set of processors.

^4^http://www.infinibandta.org

^5^http://www.open-mpi.org

^6^http://www.ugr.es/~jfernand/mpitb.html

## Appendix

The source codes for deep unsupervised learning provided below are not tied to the specific benchmark task and they can be easily adapted to other learning problems. We provide a variety of solutions (MATLAB/Octave on a desktop PC; MATLAB and Python on a desktop with a graphic card; Octave/MPI on a multi-core cluster) and we leave it to individual researchers to choose the one that best fits their needs.

The complete source code for each implementation can be downloaded from the following website: http://ccnl.psy.unipd.it/research/deeplearning

We summarize below the main steps to follow in order to use each implementation. In order to maximize portability, all implementations assume to manipulate data stored in 3-D matrices of size *x*y*z*, where *x* is the mini-batch size, *y* is the input size (i.e., the number of visible units) and *z* is the total number of mini-batches.

We also provide instructions on how to run our codes on the popular MNIST example, which we used as a benchmark task, and on the numerosity perception problem investigated by Stoianov and Zorzi (2012).

### Graphic cards implementations

At present there are no high-level wrappers of CUDA available for Octave. We therefore exploited the proprietary MATLAB Parallel-Computing Toolbox. However, the Python implementation is completely freeware. Note that NVIDIA drivers must be properly installed on your system, along with a compatible CUDA graphic card with at least 1GB of dedicated memory.

### MATLAB

MATLAB Parallel-Computing Toolbox (v2011 or above) must be properly installed in the system.

Network and learning parameters can be directly set inside the script “*deeptrain_GPU.m*.” Running the script will train a deep belief net. At the end of the learning phase, a MATLAB file containing the results will be created into the source code directory. Note that the training data must be supplied as an additional “.mat” file (e.g., “*MNIST_data_n.mat*” for the MNIST example).

### Python

Python (tested: v2.7) and CUDAMat (tested: v1.15) must be properly installed in the system, along with the Gnumpy library (available for download from http://www.cs.toronto.edu/~tijmen/gnumpy.py) that must be present in the same folder of the source code. Network and learning parameters can be directly set inside the script “*deeptrain_GPU.py*.” Running the script will train a deep belief net. At the end of the learning phase, a “.mat” file (MATLAB/Octave) containing the results will be created into the source code directory. Note that the training data must be supplied as an additional “.mat” file (e.g., “*MNIST_data_n.mat*” for the MNIST example).

### Cluster implementation

Octave and MPITB (MPI library routines for Octave environment; download from http://www.ugr.es/~jfernand/mpitb.html) must be properly installed on the cluster (tested: Octave 3.0.5, Open-MPI 1.4.3). Network and learning parameters can be directly set inside the script “*deeptrain.m*.” Running the script will train a deep belief net. At the end of the learning phase, a “.mat” file containing the results will be created into the source code directory. Note that the training data must be supplied as an additional “.mat” file (e.g., “*MNIST_data_n.mat*” for the MNIST example).

### MNIST example

The raw MNIST dataset must be first converted into a suitable format:

1. Download the following four files from http://yann.lecun.com/exdb/mnist
  - train-images-idx3-ubyte.gz
  - train-labels-idx1-ubyte.gz
  - t10k-images-idx3-ubyte.gz
  - t10k-labels-idx1-ubyte.gz
2. Unzip them by executing
  - gunzip train-images-idx3-ubyte.gz
  - gunzip train-labels-idx1-ubyte.gz
  - gunzip t10k-images-idx3-ubyte.gz
  - gunzip t10k-labels-idx1-ubyte.gz
  Make sure the file names have not been changed during unzipping (in particular, it can happen that “-” is replaced with “.”)
3. Convert the raw images into MATLAB/Octave format by copying them into the source code directory and running the routine “*converter.m*.”
4. Save the training set into a suitable 3-D matrix by running the routine “*makebatches.m*” (mini-batch size can be set inside that file before running it). This will produce a file called “*MNIST_data_n.mat*,” where *n* is the chosen mini-batch size.

### Visual numerosity perception example

The database of images created by Stoianov and Zorzi (2012) includes 50,000 binary images containing up to 32 rectangular objects of variable size. The “.mat” file can be downloaded from http://ccnl.psy.unipd.it/research/deeplearning

To replicate the simulations of Stoianov and Zorzi (2012), the deep network should have two hidden layers of 80 and 400 units, respectively (for further details, see Supplementary Materials of Stoianov and Zorzi, 2012).
